# Combined Central Slip and Volar Plate Avulsion Fractures: A Rare Case With Nonsurgical Recovery

**DOI:** 10.1016/j.jhsg.2026.101016

**Published:** 2026-04-07

**Authors:** Shıkhalı Isgandarlı, Can Yapıcı, Cengiz Çabukoğlu

**Affiliations:** ∗ Orthopedic and Traumatology Specialist, Central Hospital Kozyatağı, İstanbul, Türkiye

**Keywords:** Central slip injury, Conservative treatment, Finger trauma, Proximal interphalangeal joint, Volar plate avulsion fracture

## Abstract

The coexistence of central slip and volar plate avulsion fractures in the same digit is uncommon and has been described primarily in isolated case reports, and current management approaches are derived mainly from isolated case reports rather than established guidelines.^1^^,^^2^ We report a case of a 26-year-old right-handed man who presented 45 days after finger trauma sustained during basketball. Clinical and radiographic evaluation demonstrated concurrent dorsal (central slip) and volar (volar plate) avulsion fractures at the proximal interphalangeal joint without instability or dislocation. The patient was treated conservatively with staged splinting and early controlled mobilization. Despite persistent radiographic fracture gaps at follow-up, the patient achieved full, pain-free range of motion without functional limitation at 6 months. This case highlights that even with delayed diagnosis, nonsurgical management can result in excellent functional outcomes when joint stability is preserved, and radiographic findings do not necessarily correlate with clinical recovery.

Avulsion injuries of the proximal interphalangeal (PIP) joint most commonly affect either the dorsal extensor mechanism or the volar stabilizing structures, each producing distinct patterns of instability and deformity.^3^^,^^4^ Central slip avulsions primarily compromise active PIP extension and predispose to boutonnière deformity,^5^ whereas volar plate avulsions destabilize the volar restraint and promote hyperextension and swan-neck deformity.^6^

These structures function as biomechanically opposing stabilizers, making simultaneous disruption uncommon and potentially challenging to manage.

When these injuries occur in isolation, well-established treatment algorithms exist; however, when both dorsal and volar stabilizers are injured in the same joint, optimal management becomes uncertain.^1^ This uncertainty is further magnified in delayed presentations, where adaptive soft-tissue remodeling and altered joint biomechanics may decouple radiographic appearance from functional behavior.

We present a delayed case of combined central slip and volar plate avulsion fractures managed nonoperatively, incorporating quantitative radiographic measurements, magnetic resonance image (MRI)-based soft-tissue assessment, and functional follow-up, to highlight how biological healing and clinical recovery may diverge from radiographic findings.

## Case Presentation

**Figure 1:**
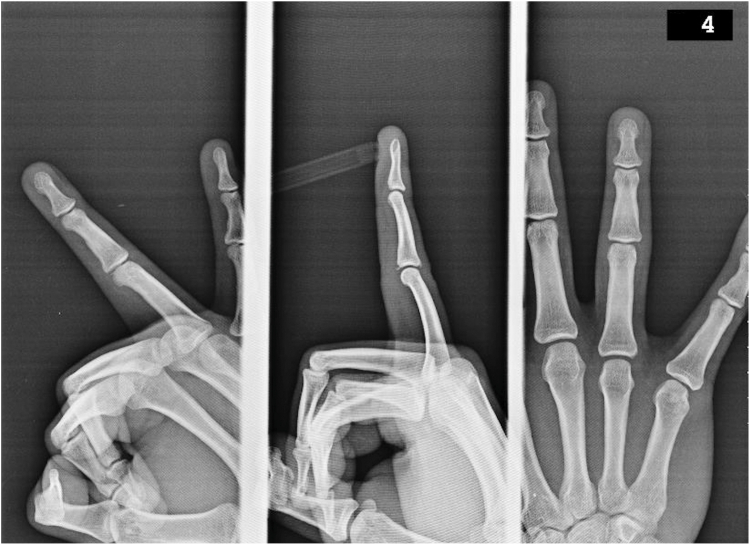
Radiograph demonstrating dorsal avulsion fracture (central slip) and volar fragment (volar plate) of the same finger.

Written informed consent for publication of clinical details and images was obtained from the patient. A 26-year-old right-handed male dentist presented with pain and swelling in his right ring finger after sustaining an injury while playing basketball. The patient reported that the ball struck the tip of his finger, causing immediate pain. Despite these symptoms, he continued his daily activities, including clinical work, for approximately 45 days before seeking medical care. Despite the patient’s initial report of isolated volar pain, physical examination revealed point tenderness over both the volar and dorsal aspects of the PIP joint, and the patient was unable to perform full flexion. The joint was stable to radial and ulnar stress. Initial radiographs demonstrated small-fragment avulsion fractures at the dorsal and volar bases of the middle phalanx of the right ring finger—corresponding to the insertion points of the central slip and volar plate, respectively—without evidence of PIP joint displacement (Fig. 1). Based on clinical and radiographic findings, a diagnosis of posttraumatic central slip and volar plate avulsion fractures was made.

**Figure 2:**
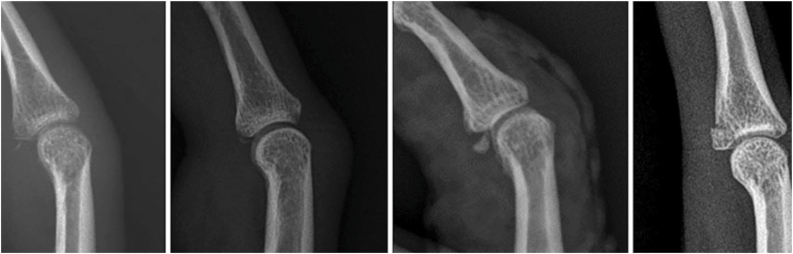
Lateral radiograph showing a sliver-shaped volar plate avulsion fragment at the base of the middle phalanx. Volar plate avulsion fractures may appear as a dot, sliver, triangle, or rhomboid fragment on lateral views.

**Figure 3:**
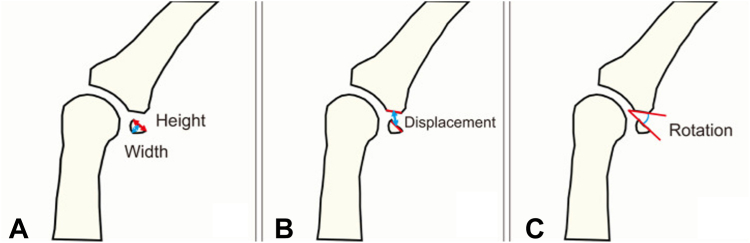
Measurement of volar plate fragment width and height on the lateral radiograph for surface area calculation (fragment size = width × height).

Volar plate avulsion fractures may appear as a dot, sliver, triangle, or rhomboid fragment on lateral views (Figs. 2 and 3).^7^ The volar plate avulsion fragment was sliver-shaped. On lateral radiography of the right ring finger, the volar fragment measured 1.7 mm in width and 1.3 mm in height, corresponding to a calculated surface area of 2.21 mm^2^. The fragment was displaced by 1.3 mm with 72° of rotation and involved approximately 10% of the PIP joint articular surface. The dorsal (central slip) fragment displacement was 0.9 mm.

**Figure 4:**
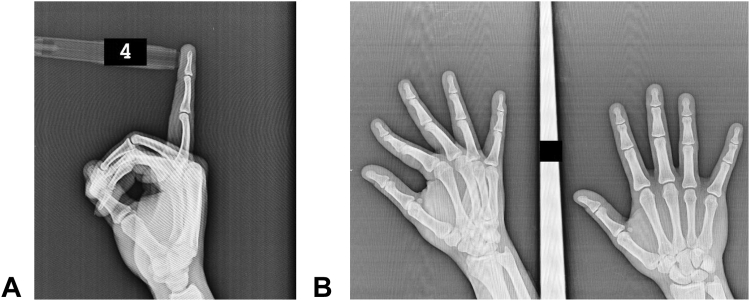
Coban wrap application in full extension with resolution of volar tenderness at a 10-day follow-up.

Initially, the PIP joint was immobilized in approximately 15° of flexion using a Coban wrap and splint. At the follow-up visit 10 days after initial immobilization, the swelling had resolved, volar tenderness was absent, and the patient reported dorsal pain only at the end range of PIP flexion. A new Coban wrap was applied, maintaining both the PIP and distal interphalangeal (DIP) joints in full extension (Fig. 4).

**Figure 5:**
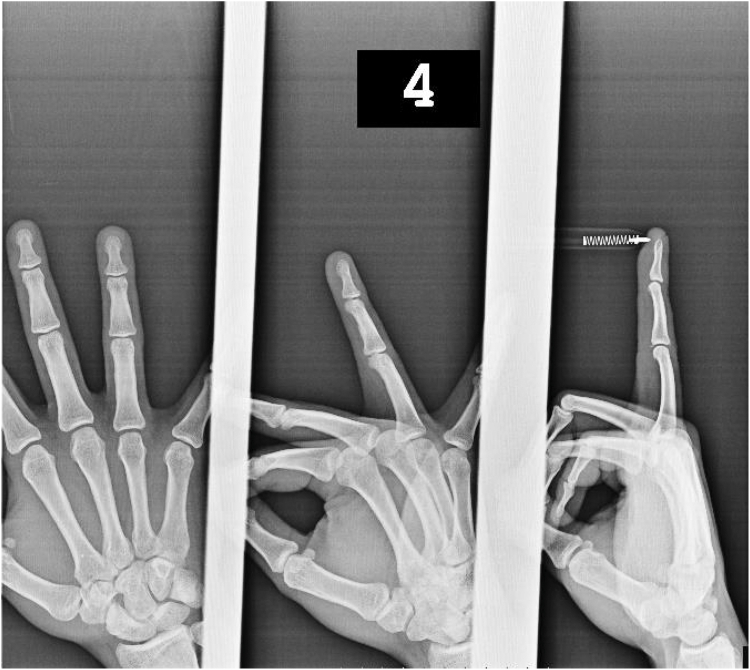
Control lateral radiograph of the right fourth finger obtained prior to application of the 30° flexion circular plaster cast.

**Figure 6:**
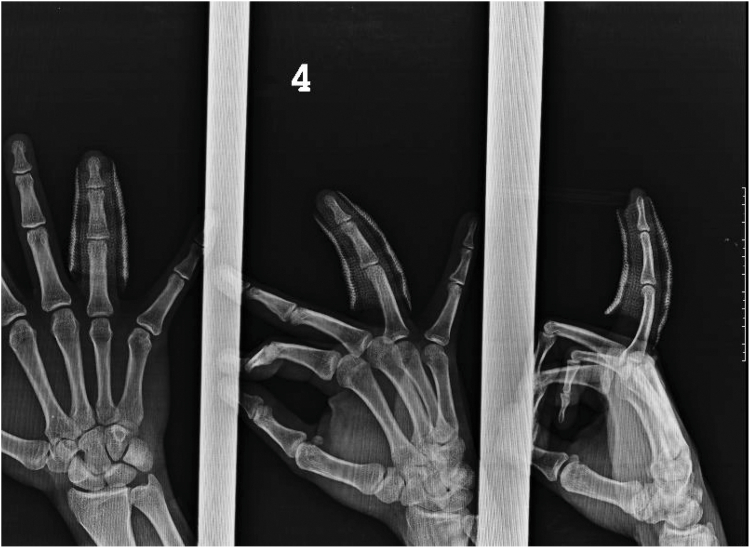
Two-week follow-up x-ray showing persistent fracture gaps

Approximately 1 week later, because of increased pain from repetitive hand use at work, a control radiograph was obtained (Fig. 5). Subsequently, a circular plaster cast was applied with the PIP joint in 30° of flexion to ensure rigid immobilization. The radiograph was obtained 2 weeks after cast application and showed a persistent gap at both the volar plate and central slip avulsion sites (Fig. 6). Following the removal of the plaster cast, clinical assessment revealed that the patient had regained a full range of motion, and further immobilization was deemed unnecessary.

**Figure 7:**
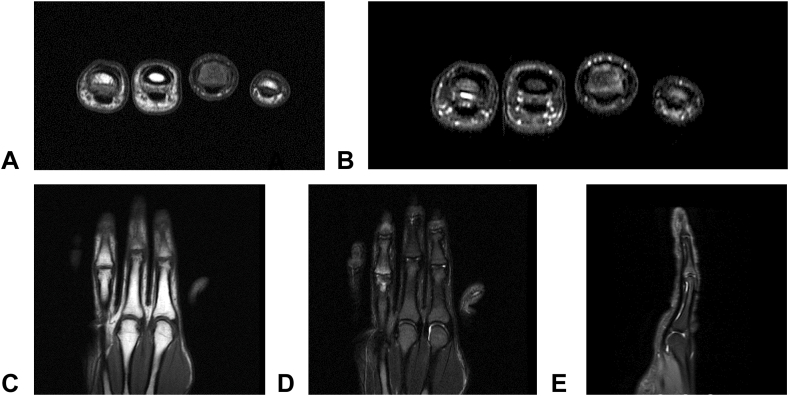
MRI demonstrating intact collateral ligaments and continuity of both the dorsal (central slip) and volar (volar plate) fragments.

**Figure 8:**
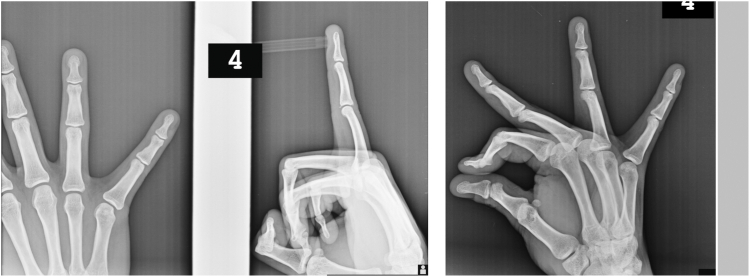
Six-month follow-up radiograph: persistent fracture gap, but full ROM and asymptomatic. ROM, full range of motıon.

A follow-up MRI was obtained after completion of treatment using coronal and axial spin-echo T1-weighted and coronal, sagittal, and axial fat-suppressed fast spin-echo T2-weighted sequences (Fig. 7). The images demonstrated intact collateral ligaments and continuity of both the dorsal (central slip) and volar (volar plate) fragments without soft-tissue disruption. At the 6-month follow-up, the patient had a full, pain-free range of motion (Video 1), whereas plain radiography demonstrated a persistent fracture gap without functional impairment (Fig. 8). Measurements across the volar fracture gap demonstrated a displacement of 1.3 mm with 69° of rotation, with fragment dimensions of 1.5 × 1.0 mm, corresponding to a surface area of 1.50 mm^2^, compared with 2.21 mm^2^ at presentation. Across the dorsal (central slip) fracture gap, the fragment displacement measured 0.7 mm.

Clinical examination demonstrated that the PIP joint was stable to radial and ulnar stress testing, with no evidence of dorsal or volar instability. Management followed a structured, stepwise treatment protocol guided by clinical and functional criteria (Table).

**Table.** Therapeutic Timeline for Nonsurgical Management

Date	Clinical Status	Splinting	Pain Status	Range of Motion
December 25, 2024	Initial diagnosis after a 45-d delay. Dorsal and volar avulsion fractures confirmed.	Coban wrap with approx. 15° PIP flexion	Severe pain (both volar and dorsal)	Restricted because of pain
January 4, 2025	A 10-d follow-up. Volar pain resolved; mild dorsal tenderness remained.	Static splint in full PIP and DIP extension	Volar pain resolved; dorsal pain persists at end range	Improved but not full
January 13, 2025	Increased pain during hand use; splint modified.	PIP joint at 30° flexion (plastic finger cast)	Recurrent dorsal pain with activity	Improving, mild limitation with use
January 27, 2025	Radiographic gap persists; splint discontinued. Active motion allowed.	Finger cast removed	Pain resolved	Full ROM achieved
May 2025	A 5-mo follow-up: full ROM and no pain despite persistent fracture gap on x-ray.	No splint	Pain-free	Full ROM maintained

DIP, distal interphalangeal; ROM, full range of motıon; PIP, proximal interphalangeal.

## Discussion

Although combined central slip and volar plate avulsion injuries have been previously reported, most published cases emphasize the rarity of this pattern rather than the biological behavior of healing. This combination creates competing biomechanical demands in management, as it is usually associated with forces acting in opposite directions, including axial loading followed by sudden hyperextension or hyperflexion.^2^^,^^8^^,^^9^ Our case provides additional observational insight by showing that radiographic fracture gaps may persist despite progressive biological remodeling and favorable functional recovery. Quantitative reduction in fragment surface area over time, rather than the mere presence or absence of a fracture line, proved to be a more meaningful indicator of healing in this patient.

The opposing biomechanical roles of the dorsal extensor mechanism and the volar stabilizing structures create potential therapeutic tension when both are injured. Conventional protocols for these injuries in isolation prescribe diametrically opposite immobilization strategies—extension for central slip injuries and flexion for volar plate injuries.^10^ In literature, when both structures are injured, the general consensus is to prioritize the central slip to prevent a permanent boutonnière deformity, as volar plate injuries are often more forgiving with early controlled motion. In delayed presentations, however, strict adherence to either protocol may be secondary to restoring balanced joint kinematics and allowing controlled motion to facilitate adaptive remodeling.

Although computed tomography provides superior visualization of cortical bone healing, MRI was preferred because it allowed for the simultaneous evaluation of bony continuity and the integrity of the key soft-tissue stabilizers of the PIP joint, which primarily determine functional outcome in these injuries.^11^ Despite persistent radiographic gaps in this case, MRI confirmed preserved continuity of both dorsal and volar soft-tissue stabilizers. Similar to findings in chronic avulsion cases, our case supports the possibility that functional stability may be maintained by fibrous continuity rather than complete bony union. The reduction in fragment surface area over 6 months (from 2.21–1.50 mm^2^) indicates an active biological remodeling process even in the absence of cortical bridging.^12^

The dissociation between radiographic appearance and clinical outcome observed here has important implications for clinical decision-making. Reliance on fracture lines alone may prompt unnecessary surgical intervention, whereas integrating clinical stability, range of motion, quantitative radiographic remodeling, and soft-tissue integrity provides a more accurate assessment of healing.^13^

This case is distinguished by 4 key aspects: the markedly delayed presentation; quantitative radiographic assessment of fragment displacement, rotation, and area over time; MRI-based confirmation of preserved soft-tissue stabilizers; and the demonstrated dissociation between radiographic appearance and clinical recovery. Together, these features offer additional insight into understanding biological and functional healing in combined dorsal and volar PIP joint avulsion injuries. In conclusion, this case supports the perspective that functional and biological recovery, rather than radiographic union alone, may help guide the management of combined dorsal and volar PIP joint avulsion injuries, particularly in delayed presentations.

## Patient Consent

Written informed consent was obtained from the patient for publication of this case report and accompanying images.

## Conflıcts of Interest

No benefits in any form have been received or will be received related directly to this article.

## Supplementary Data

Video 1 At the 6-month follow-up, the patient had a 73° range of motion that was full and pain-free.

## References

[1] Alotaibi A.S., Almarshad F.A., Alzahrani A.M. (2021). Simultaneous central slip and volar plate injuries at PIP joint: a novel therapeutic approach. Plast Reconstr Surg Glob Open.

[2] Lo I., Richards R.S. (1995). Combined central slip and volar plate injuries at the PIP joint. J Hand Surg Br.

[3] Imatami J., Hashizume H., Wake H., Morito Y., Inoue H. (1997). The central slip attachment fracture. J Hand Surg Eur Vol.

[4] Pang E.Q., Yao J. (2018). Anatomy and biomechanics of the finger proximal interphalangeal joint. Hand Clin.

[5] Binstead J.T., Tafti D., Hatcher J.D. (Published online August 7, 2023). Boutonniere deformity. StatPearls.

[6] Caviglia D., Ciolli G., Fulchignoni C., Rocchi L. (2021). Chronic post-traumatic volar plate avulsions of the finger proximal interphalangeal joint: a literature review of different surgical techniques. Orthop Rev.

[7] Lee S., Jang S.J., Jeon S.H. (2020). Factors related to failure of conservative treatment in volar plate avulsion fractures of the proximal interphalangeal joint. Clin Orthop Surg.

[8] Bowers W.H., Wolf J.W., Nehil J.L., Bittinger S. (1980). The proximal interphalangeal joint volar plate. I. An anatomical and biomechanical study. J Hand Surg Am.

[9] Viegas S.F. (Published online 1997). Hand Surgery Study Guide.

[10] Freiberg A., Pollard B.A., Macdonald M.R., Duncan M.J. (2006). Management of proximal interphalangeal joint injuries. Hand Clin.

[11] Clavero J.A., Alomar X., Monill J.M. (2002). MR imaging of ligament and tendon injuries of the fingers. Radiographics.

[12] Povlsen B., Singh R. (2010). Outcome of late presentation of injuries of the volar plate of the proximal interphalangeal joint. Scand J Plast Reconstr Surg Hand Surg.

[13] Calfee R.P., Sommerkamp T.G. (2009). Fracture-dislocation about the finger joints. J Hand Surg.

